# BP230‐Type Bullous Pemphigoid is a Rare Subset of Bullous Pemphigoid Associated With a Favorable Prognosis: A Retrospective Analysis From a Tertiary Referral Center

**DOI:** 10.1111/ijd.70186

**Published:** 2025-11-30

**Authors:** Roberto Maglie, Sefano Senatore, Carlo Pipitò, Maria E. Baffa, Stefano Colabrese, Sasha Visinoni, Vincenzina Rubino, Anna Pira, Feliciana Mariotti, Marzia Caproni, Giovanni Di Zenzo, Emiliano Antiga

**Affiliations:** ^1^ Department of Health Sciences, Section of Dermatology University of Florence Florence Italy; ^2^ Molecular and Cell Biology Laboratory Istituto Dermopatico dell’Immacolata (IDI)‐IRCCS Rome Italy

Bullous pemphigoid (BP) is the most common autoimmune bullous disease. It usually presents with widespread blistering of the skin and intense pruritus; atypical presentations lacking blisters have also been reported [1]. BP is associated with autoantibodies targeting the NC16A domain of Collagen XVII (BP180) [1]. Pathogenic activity of BP230‐IgG has also been reported in BP [2]. A minority of patients have elevated serum levels of BP230‐IgG while being negative for BP180‐NC16A‐IgG in commercial enzyme‐linked immunosorbent assays (ELISAs); whether these patients constitute a different subset of BP is still debated [3].

Following approval from our institutional review board, we set up a monocentric retrospective analysis of clinical characteristics and outcomes of patients diagnosed with BP230‐type BP over a period of 3 years. Diagnosis of BP was based on IgG and/or C3 deposition at direct immunofluorescence microscopy on perilesional skin. Selected cases were BP patients showing elevated titers of BP230‐IgG and absence of BP180‐NC16A‐IgG by ELISA. To exclude potential antibody reactivity against non‐NC16A epitopes of BP180, sera from these patients were analyzed both by immunoblotting (IB) using keratinocyte extracts and by a novel in‐house ELISA based on the entire ectodomain (ECD) of BP180 [4]. Those who were negative to the latter tests were eventually classified as having BP230‐type BP.

Of 147 patients diagnosed with BP, 14 were BP180‐NC16A‐IgG negative and BP230‐IgG positive (eight females, six males; median age 79.4 years). Eight presented with classic BP, while the remaining six had atypical nonbullous variants. Interestingly, IB on these 14 sera showed IgG positivity to BP180 antigen in nine cases (Figure 1a), four of which also had positive results in ECD‐BP180 ELISA (Figure 1b).

**FIGURE 1:**
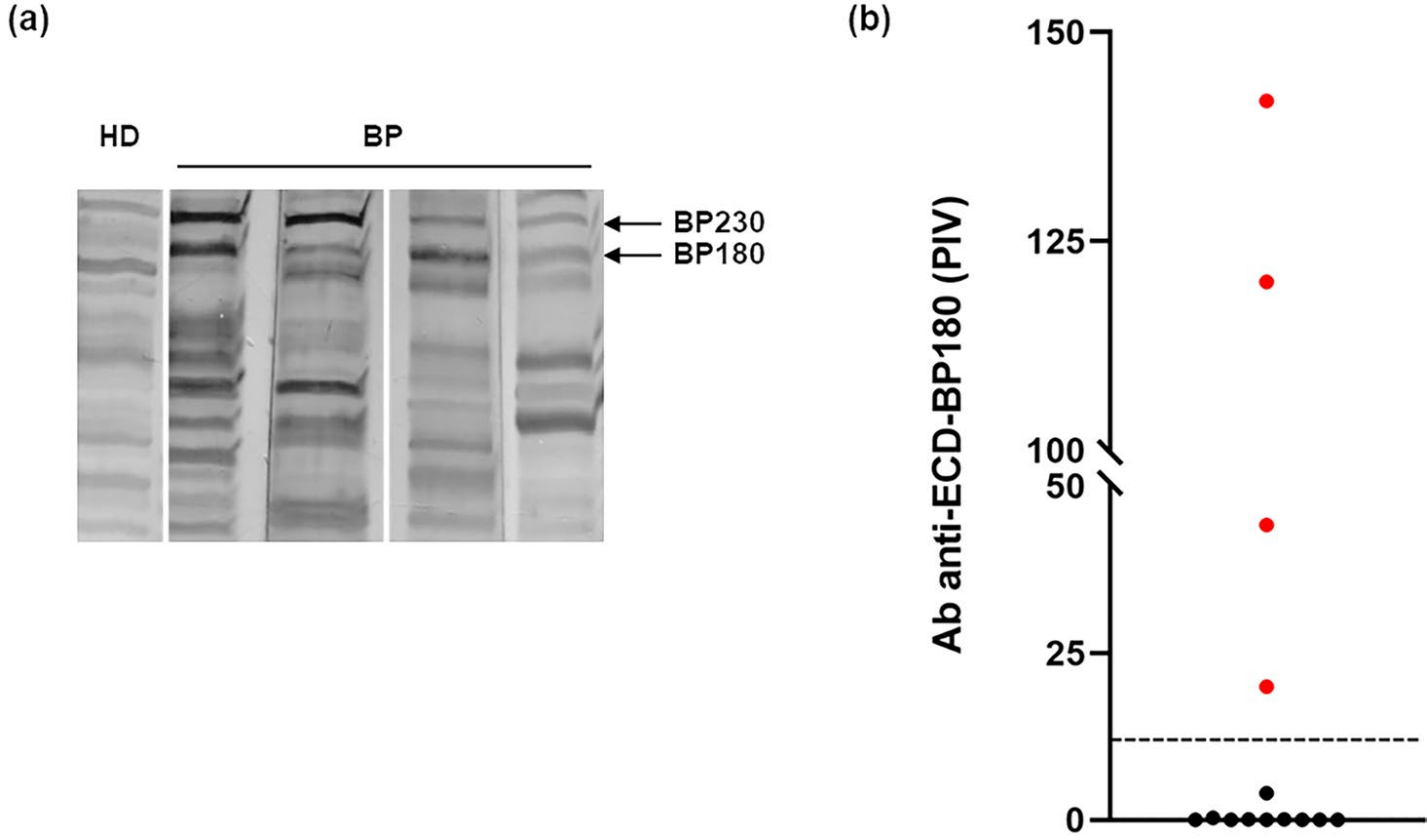
Immunological reactivity of bullous pemphigoid sera by immunoblotting and ELISA. (a) Four representative BP sera, reacting to BP230 and BP180, are shown in immunoblotting filters. Healthy donor (HD) serum, as negative control, is also indicated. (b) Graphical representation of IgG reactivity by ECD‐BP180 ELISA of 14 bullous pemphigoid (BP) sera that were negative using commercial BP180‐NC16A ELISA. Red dots indicate the samples that lie above the cut off value (dashed black line); PIV, pemphigoid index value, represents the standardization of extinction data on the positive and negative internal standards.

Indeed, only five patients (all females, median age 86 years, range 80–94) were classified as BP230‐type BP. Except for one, all presented with nonbullous BP (localized BP (Figure 2a), prurigo‐like BP (Figure 2b), eczematous BP (Figure 2c), and one had an urticarial pre‐bullous stage). None had mucosal involvement.

**FIGURE 2:**
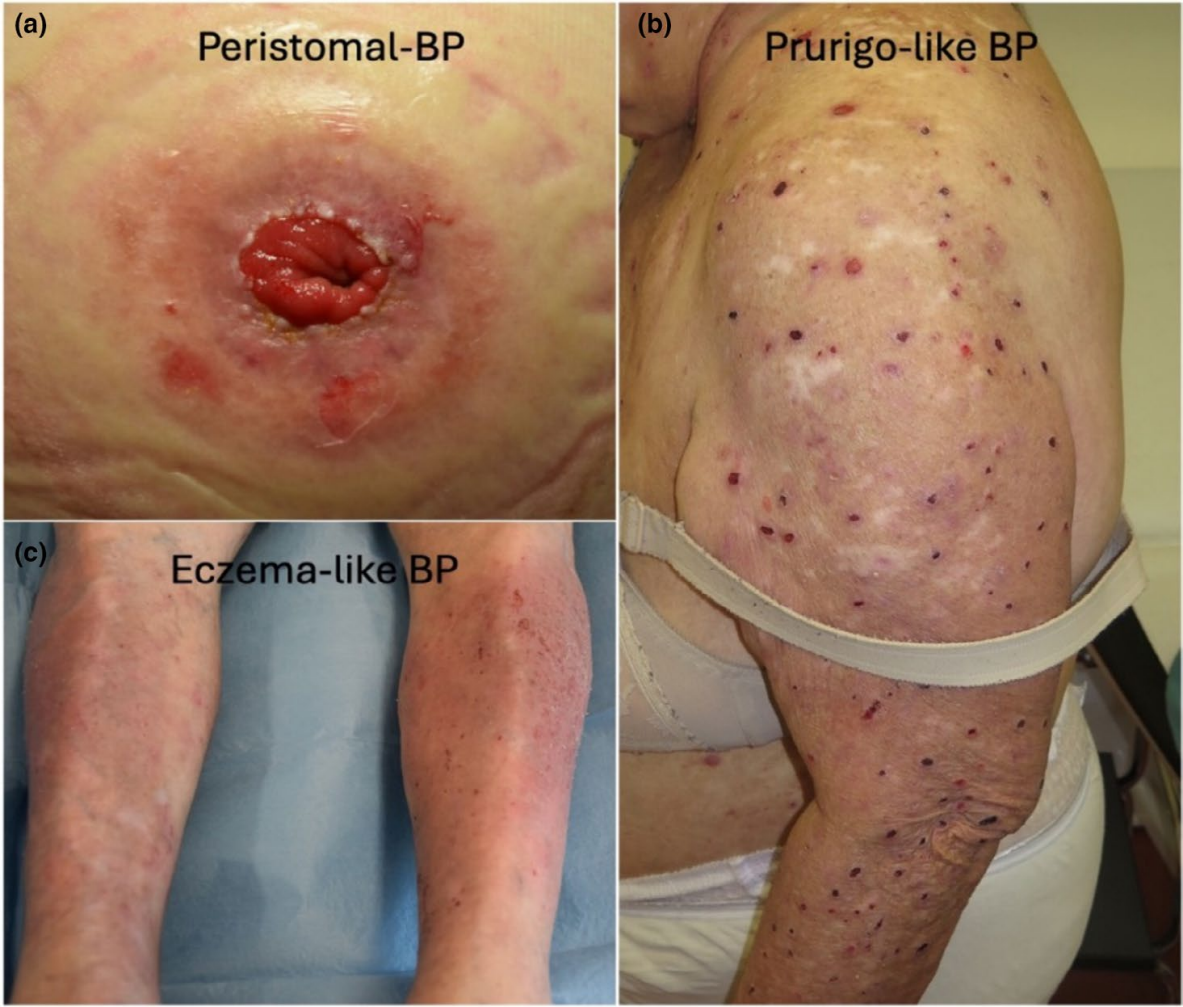
Representative images of patients with BP230‐type BP: (a) peristomal BP; (b) prurigo‐like BP; (c) eczema‐like BP.

All patients received first‐line treatment with topical steroids and oral prednisone (0.3 mg/kg/day or lower), which in one case was paired with oral doxycycline. None required adjuvant immunosuppressive treatments, and disease remission was achieved in 1 month for four patients and 2 months for one patient. During a 36‐month follow‐up period, two patients experienced one relapse, after 4 and 6 months, respectively, and were successfully re‐treated with a tapering course of oral prednisone.

The key message of our study is double: first, BP230‐type BP is rare (3.4% in our series). Accordingly, most of the serum samples positive for BP230‐IgG and negative for BP180‐NC16A‐IgG revealed antibody reactivity against either the full‐length BP180 protein or its ECD. Second, “true BP230‐type BP” is likely to present with nonblistering variants. This finding aligns with experimental studies linking the pathogenicity of BP230‐IgG with erythema and pruritus rather than blisters [5].

In our series, BP230‐type BP was preferentially associated with female sex, but not with specific comorbidities, such as neurological diseases. Regarding prognosis, BP230‐type BP ran a benign clinical course, confirming the positive prognostic implication of BP180‐IgG negativity in BP.

In conclusion, from a clinical point of view, patients who are BP230‐IgG positive but BP180‐NC16A‐IgG negative by commercial ELISAs and present with atypical non‐bullous variants are presumed to suffer from a “true BP230‐type BP”, and thereby frontline aggressive therapies may be avoided. Here, the use of the ECD‐BP180 ELISA proves to be a useful tool to identify this subset of patients.

Limitations of this study include its retrospective design and small sample size, which aligns with previous data in the literature [3].

Further studies aimed at elucidating the role of BP230 in the pathogenesis of BP are welcomed.

## Funding

The authors have nothing to report.

## Conflicts of Interest

The authors declare no conflicts of interest.

## Data Availability

The data that support the findings of this study are available from the corresponding author upon reasonable request.
